# Non-apoptotic programmed cell deaths in diabetic pulmonary dysfunction: the new side of advanced glycation end products

**DOI:** 10.3389/fendo.2023.1126661

**Published:** 2023-10-26

**Authors:** Yimin Dai, Shuang Zhou, Lin Qiao, Zhao Peng, Jiuliang Zhao, Dong Xu, Chanyuan Wu, Mengtao Li, Xiaofeng Zeng, Qian Wang

**Affiliations:** Department of Rheumatology and Clinical Immunology, Chinese Academy of Medical Sciences and Peking Union Medical College, National Clinical Research Center for Dermatologic and Immunologic Diseases (NCRC-DID), Ministry of Science and Technology, State Key Laboratory of Complex Severe and Rare Diseases, Peking Union Medical College Hospital (PUMCH), Key Laboratory of Rheumatology and Clinical Immunology, Ministry of Education, Beijing, China

**Keywords:** diabetic pulmonary dysfunction, advanced glycosylation end products, non-apoptotic programmed cell deaths, inflammation, oxidative stress

## Abstract

Diabetes mellitus (DM) is a chronic metabolic disorder that affects multiple organs and systems, including the pulmonary system. Pulmonary dysfunction in DM patients has been observed and studied for years, but the underlying mechanisms have not been fully understood. In addition to traditional mechanisms such as the production and accumulation of advanced glycation end products (AGEs), angiopathy, tissue glycation, oxidative stress, and systemic inflammation, recent studies have focused on programmed cell deaths (PCDs), especially the non-apoptotic ones, in diabetic pulmonary dysfunction. Non-apoptotic PCDs (NAPCDs) including autophagic cell death, necroptosis, pyroptosis, ferroptosis, and copper-induced cell death have been found to have certain correlations with diabetes and relevant complications. The AGE–AGE receptor (RAGE) axis not only plays an important role in the traditional pathogenesis of diabetes lung disease but also plays an important role in non-apoptotic cell death. In this review, we summarize novel studies about the roles of non-apoptotic PCDs in diabetic pulmonary dysfunction and focus on their interactions with the AGE–RAGE axis.

## Introduction

Diabetes mellitus (DM) has long been considered a systemic disease that is closely related to autoimmune disorders (1). Pulmonary involvement of DM patients was first mentioned in the 1970s (2, 3). From then on, multiple cases and retrospective studies linking pulmonary diseases such as chronic obstructive pulmonary disease (COPD) and lung fibrosis to DM have continuously been reported (4–10) with little clear and direct evidence obtained and contradictory conclusions appearing in different studies (11). Given that pulmonary function indexes are significant indicators of lung condition, clinical researchers have investigated its correlation with DM. In 1985, researchers summarized characteristics of the pulmonary function of DM patients documenting a significant decrease in forced vital capacity (FVC) and timed vital capacity (TVC), as well as a decrease in forced expiratory volume in 1 second (FEV1) and diffusion lung capacity for carbon monoxide (CO) (DLCO) in male DM patients (12). Subsequently, multiple studies have compared the pulmonary function of DM patients and healthy controls. Systemic reviews found bidirectional conclusions, but the decrease of FVC and FEV1 as well as the negative association between pulmonary function and severity of DM was frequently reported (13–15).

Although damage to pulmonary function in DM patients is frequently observed and analyzed, the possible underlying mechanisms are less reviewed in comparison (11). Previous perspectives on this matter have primarily focused on metabolic dysfunction, inflammation reaction, neurological deficit, and their combined effects (11). The impaired glucose metabolism in DM patients may lead to the deposition of glycolyzed serum proteins in the vascular system (16). Due to their rich capillary network, the lungs are vulnerable to diabetic microangiopathy, which can cause impaired pulmonary ventilation function (17). Additionally, the continuous pro-inflammatory response and damage to the autonomic nervous system of the lungs have also been mentioned (18, 19). While these traditional perspectives are convincing to some extent, more innovative mechanisms are expected to be uncovered.

In recent years, researchers have extensively studied programmed cell deaths (PCDs) in multiple systems and organs and have identified various pathogenic mechanisms of multiple diseases, among which the non-apoptotic ones have been newly identified (20–22). Although apoptosis has long been considered the most conservative form of PCD, non-apoptotic PCDs (NAPCDs) have gained increasing interest. These non-apoptotic PCDs possess unique characteristics that can compensate for failed apoptosis or independently contribute to disease development. Therefore, they have been considered potential new breakthroughs in the study of diseases (23). Non-apoptotic PCDs that have been commonly mentioned mainly include autophagic cell death, necroptosis, pyroptosis, ferroptosis, and the recently identified copper-induced cell death (21, 24–26).

Non-apoptotic PCDs have been found to have correlations with diabetes and its complications, primarily due to the activation of inflammatory reactions and the roles of inflammasomes (27). Non-apoptotic PCDs have been shown to have significant effects on diabetic cardiovascular disease and nephropathy (20, 28). However, considering the overlapping roles of non-apoptotic PCDs in pneumopathy and diabetes, it is necessary to investigate the possible effects of non-apoptotic PCDs on diabetic pulmonary dysfunction and its associations with traditional pathogenic mechanisms.

In this essay, we will review recent studies on the roles of non-apoptotic PCDs including autophagic cell death, necroptosis, pyroptosis, ferroptosis, and copper-induced cell death in the impairment of pulmonary function in DM and their interactions with traditional mechanisms especially with the advanced glycation end product (AGE)–RAGE axis. Previous studies have focused on mechanisms related to inflammation and metabolism dysfunction as well as their combined effects. By exploring the molecular level of pulmonary damage associated with DM, this review aims to provide novel perspectives and insights.

## Traditional roles of the AGE–RAGE axis contributing to pulmonary dysfunction in diabetes

Abnormal glucose metabolism is closely related to DM, which involves complex organic reactions and the production of AGEs. These products have long been validated to be associated with various diabetic complications (29, 30). Specifically, the produced AGEs further accumulate and cause various lesions and tissue damage (29). The lung is a possible target organ of AGEs due to its rich constitution of capillaries, collagen, and elastin fibers that are prone to deposit AGEs (31, 32).

### Production and signaling of AGEs

Non-enzymatic glycation reactions (Maillard reaction) occur in DM patients due to hyperglycemic conditions and largely result in the production of endogenous AGEs (33). Previous studies have already figured out the increase in the accumulation of AGEs in COPD patients (34–36). In addition, significantly increased levels of AGEs, as detected through skin autofluorescence (SAF), have also been observed in DM patients who exhibit only decreased FVC, FEV1, and DLCO yet no specific pulmonary diseases (37, 38), indicating that damage occurs before the onset of any diseases. The production of AGEs can result in a series of downstream reactions and can contribute to the development of various pathogenic states (39). One of the pulmonary reactions often associated with AGEs turns out to be the AGE receptor (RAGE) on alveolar epithelial cells (40, 41). An improvement in lung compliance and diminishing adherence of cells to matrix proteins were found after blocking the RAGE, which could partially explain the deficit in pulmonary function (42, 43).

The RAGE–AGE reaction has been identified to play a role in several classical signaling pathways and has been linked to lung homeostasis (44). Endogenous AGEs cross-link with extracellular collagen and hemoglobin extracellular (45), while intracellular responses mainly depend on pathways relevant to nuclear factor kappa-B (NF-κB) and Janus kinase (JAK)-signal transducer and the activator of the transcription (STAT) pathway reaction (44). Possible mechanisms behind this reaction may include increased vascular permeability, activation of inflammatory state, production of oxidative stress, angiogenesis, and dysfunction of the pulmonary epithelium (40, 44, 46).

### The AGE–RAGE axis in angiopathy signaling

The abundant vasculature in the lungs has long been considered a crucial target leading to diabetic lung injury, and pulmonary vascular disease (PVD) is a significant cause of damage to pulmonary function (47–49). In the early preclinical stages of diabetes, the subclinical vascular effects were found to be mainly manifested as dysfunction of the endothelium of conductance and resistance arteries, which is associated with a decrease in nitric oxide (NO) bioavailability and abnormal production of reactive oxygen species (ROS) in hyperglycemic states (50, 51). Additionally, the destruction of endothelial integrity due to pulmonary microvascular injury was found to mainly affect CO transfer capacity (52). This finding is consistent with the significant decrease in DLCO in DM patients observed in clinical studies (49).

The RAGE mentioned above is highly expressed in pulmonary small arteries and arteriolar capillaries (53, 54). Researchers have found that the RAGE–AGE reaction results in the upregulation of the vascular cell adhesion molecule-1 (VCAM-1) by increasing NF-κB. This can increase the permeability of inflammatory cells across vascular endothelium and accelerate pulmonary angiopathy (41, 55). Furthermore, NO is involved in the above pathway as well; the AGEs on endothelium may downregulate endothelial nitric oxide synthase (eNOS) and reduce the production of NO (56). This is possibly correlated with low shear stress resulting from atherosclerosis vessels, and the decreased NO further leads to activation of NF-κB (57). These signaling pathways intersect in a network and are involved in multiple other pathophysiological effects such as inflammation and oxidative stress.

### AGEs alter pulmonary structures

In addition to several direct effects on vascular pathogenesis, AGEs have been found to stimulate the expression of transforming growth factor-β (TGF-β), which then facilitates the synthesis of collagen, laminin, and fibronectin in the extracellular matrix (ECM) (58). Hyperglycemia is known to significantly induce epithelial-to-mesenchymal transition (EMT) of alveolar epithelial cells by upregulating TGF-β, leading to lung fibrosis and decreased pulmonary function indexes such as FVC and total lung capacity (TLC) (59–61). The accumulation of extracellular collagen can deposit in the lung and chest wall, causing restrictive damage to pulmonary function (62). Moreover, AGEs can increase the stiffness of the vasculature by cross-linking elastin and collagen, enlarging the area of the ECM (63). AGEs can also bind to lipids, and the produced glycated low-density lipoprotein (LDL) has been shown to reduce the production of NO and mediate endothelial homeostasis (64). These extracellular changes in tissues result from glycation and AGE effects, forming the glycation network with the previously mentioned intracellular signaling, leading to pulmonary dysfunction (65).

Changes in the pulmonary interstitium, including the thickening of the alveolar epithelium and basal lamina of pulmonary capillaries, can significantly affect the viscoelasticity of lung tissue and cause alveolar collapse (66). These alterations also lead to decreased perfusion of pulmonary capillaries (67). Additionally, the apoptosis of alveolar epithelium cells as well as capillary endothelium cells can result in damage to the alveolar capillary network, destruction of pulmonary tissue, and the development of lesions such as emphysema (68). Possible relevant signaling pathways may involve c-Jun N-terminal kinase (JNK) and NF-κB (69). These injuries to alveoli and capillaries further result in a mismatch of ventilation perfusion and a reduction of diffusion capacity, that is, DLCO loss in pulmonary function (70). On a larger scale, the accumulation of collagen in the pulmonary interstitium and chest wall due to glycation in DM can rigidify the lung and rib cage, adding to the restrictive pathophysiology (62, 71).

### Oxidative stress and inflammation reaction

Glycation and its products can stimulate tissues and organs in DM patients to respond abnormally, resulting in sustained oxidative stress due to the generation of ROS and subsequent inflammatory state with an attack of proinflammatory cytokines (46). This has long been considered a significant pathogenic mechanism for the development of DM complications in various organs and systems, including the respiratory system (30). The oxidative stress can exacerbate the inflammatory response, leading to tissue damage and further generation of more ROS and oxidative stress in turn. Such a vicious cycle also exists in the pathogenesis of pulmonary dysfunction (72).

### Production of ROS in diabetes

Multiple studies have already identified the significant role of ROS in the development of pulmonary diseases and lesions, such as asthma, COPD, and lung fibrosis (73–76). The excess production of ROS disrupts the redox balance in lung tissues, leading to serious consequences such as telangiectasia and even pulmonary function failure (77). DM has been identified to be closely associated with endogenous ROS, largely resulting from AGE–RAGE reactions that activate the reduced form of nicotinamide adenine dinucleotide (NADH) oxidase to synthesize more ROS (78–80). The high production of ROS in turn facilitates more AGEs, leading to another vicious cycle (33, 46).

The destruction of pulmonary tissues by ROS is a serious issue that results in damage to multiple constituents, including DNA, lipids, and proteins (81–83). Moreover, ROS-induced mitochondrial injuries in the lungs have also been suspected to play roles in diabetic pulmonary dysfunction (80, 84). The decrease in the sirtuin 3 (SIRT3) protein in mitochondria under ROS stimulation from the diabetic lung has been found to support mitochondria injuries and possible SIRT3-dependent injury pathways as well (80, 85). However, as an important ROS producer, mitochondria generating excessive ROS under induction of hyperglycemia can also contribute to cellular injuries. An increased level of ROS has been identified to cause abnormal opening of the mitochondrial permeability transition pore (MPTP), followed by the release of cytochrome *c*, caspase activation, generation of more ROS, and finally cell apoptosis (86, 87). Furthermore, researchers also have figured out that the reaction between NO and ROS produces toxic reactive nitrogen species, which further results in vasoconstriction and endothelial injury (88). The involvement of oxidative stress in the formation of pulmonary angiopathy is also a significant constitution in the pathogenesis of pulmonary dysfunction.

### Inflammatory response

The downstream inflammatory response resulting from ROS is also considered an important pathogenesis in pulmonary dysfunction. One of the central pathways is the NF-κB signaling pathway, which has been identified to regulate the expression of proinflammatory cytokines (89). Relevant cytokines include several interleukins (ILs; IL-1β, IL-4, IL-5, IL-6, IL-8, and IL-13) as well as tumor necrosis factor-α (TNF-α) and mucin. Together, these cytokines contribute to the formation of an inflammatory state and further lead to pulmonary dysfunction and even disease development (72, 90). Inflammatory responses modulated by NF-κB also involve inducible nitric oxide synthase (iNOS), resulting in excessive nitrosative stress, i.e., the formation of elevated levels of peroxynitrite (91–93). This has been linked to pulmonary vascular injuries (94). Moreover, followed by NF-κB activation, the upregulation of mitogen-activated protein kinases (MAPKs) is a result of the AGE–RAGE reaction (93). In addition, NF-κB also induces ROS production by activating NADPH oxidase through AGE–RAGE, which is independent of MAPK regulation (92). Research on diabetic rats has found significantly increased levels of NF-κB, along with upregulation of downstream iNOS in lung tissue (95). Conversely, inhibition of NF-κB can reduce the generation of proinflammatory cytokines IL-6 and TNF-α in respiratory tract epithelium, alleviating lung injury (95, 96). These findings provide strong evidence supporting the inflammatory response mediated by NF-κB in pulmonary dysfunction pathogenesis.

Apart from the classical NF-κB pathway, the JAK-STAT pathway has also been revealed to mediate inflammatory responses under the AGE–RAGE reaction, particularly the JAK2/STAT3 signaling pathway (97, 98). Animal studies have shown that negative regulation of JAK2/STAT3 can significantly relieve pulmonary epithelial injuries in response to hyperglycemia and pancreatic dysfunction while also reducing the levels of proinflammatory cytokines such as IL-6, IL-18, and TNF-α (98, 99). Activated by inflammatory cytokines such as IL-6, JAK2/STAT3 plays roles in inflammatory response (100). Furthermore, the upregulation of TGF-β via JAK2/STAT3 promotes profibrotic responses in lung tissue, leading to restrictive pulmonary dysfunction (101).

### Infection and dysregulation of immune response

It is well established that poorly controlled diabetes in DM patients can increase the risk of infection in various tissues, organs, and systems, including the respiratory system (102, 103). This greatly increases hospitalization and mortality rates. Common pathogens that cause respiratory infection include bacteria such as *Streptococcus pneumoniae*, fungi such as *Aspergillus fumigatus*, tuberculosis, influenza virus, and certain viruses that cause specific epidemics, such as H1N1, MERS-CoV, and SARS-CoV-2 (104–106). The strike of respiratory infection can be devastating. According to clinical research conducted among hospitalized cystic fibrosis (CF) patients, those with *A. fumigatus* infection had significantly reduced FEV1 compared to those without infection. Moreover, persistent infection was identified as an independent risk factor for these patients (107).

Alterations of the immune system have been found in DM and are quite possibly associated with the increased risk of pulmonary infection (108). On the one hand, the killing effects of immune cells can be largely weakened in DM due to functional defects. For example, production of the bactericidal ROS, as well as the impaired migration function, can be reduced due to injured glucose metabolism (109, 110). In addition, the treatment of DM may also contribute to reduced generation of cytokines (111). On the other hand, the immune response can be excessively activated in DM under an infection state, resulting in damage to normal tissue (112). Such double effects in diabetic immune response toward invasion of pathogens may partially explain the mechanisms of lung injury caused by infection.

## Non-apoptotic PCDs and relevant mechanisms

As types of regulated cell death differ from conservative apoptosis, non-apoptotic PCDs have become targets for intervention in diseases. Autophagic cell death, necroptosis, pyroptosis, ferroptosis, and even the newly reported copped cell death, e.g., cuproptosis, all play roles in certain diseases (23, 113). The regulation pathways of non-apoptotic PCDs were summarized in **Table 1**.

**Table 1:** The regulation pathways of different types of non-apoptotic programmed cell deaths.

Non-apoptotic cell death	No.	Pathway	Observed molecular changes	Effects	Ref.
Autophagic cell death	1	AGE/RAGE → class III PI3K/Beclin-1	Upregulation of Beclin-1	Facilitate autophagy cell death	(114)
	2	AGE/RAGE → class I PI3K/Akt/mTOR	Inhibition of class-I PI3K/Akt/mTOR	Facilitate autophagy cell death	(114)
	3	AGE/RAGE → mTOR	Inhibition of mTOR	Facilitate autophagy cell death	(115)
	4	ROS → mTOR	Upregulation of mTOR	Inhibiting autophagy cell death	(116)
Pyroptosis	5	HMGB1/RAGE → pyroptosome → caspase-1 → SIRT1 → AP-1 → VCAM-1	Upregulation of caspase-1 Upregulation of VCAM-1	Facilitate pyroptosis Facilitate adherence of monocytes and vascular damage	(78, 117–120)
	6	HMGB1/RAGE → caspase-11 → caspase-1	Upregulation of caspase-1	Facilitate pyroptosis	(121, 122)
	7	AGE/RAGE → MAPK →NF-κB → TLR → NLRP3 → caspase-1 → IL-1β and IL-18	Upregulation of caspase-1 Upregulation of IL-1β and IL-18	Facilitate pyroptosis Facilitate inflammation	(93, 122–124)
	8	HMGB1/LPS/AGEs	Exacerbate 5~7	Facilitate pyroptosis	(125)
	9	AGE/RAGE → JAK/STAT → GSDMD	Upregulation of GSDMD	Facilitate release of pro-inflammatory cytokines	(97, 126, 127)
	10	ROS → NLR P3 → caspase-1 → IL-1β	Upregulation of caspase-1 Upregulation of IL-1β	Facilitate pyroptosis Facilitate inflammation	(128)
	11	CML/HMGB1/RAGE → TLR + NF-κB	Upregulation of TLR and NF-κB	Facilitate pyroptosis	(129)
Necroptosis	12	CML/RAGE → RIPK3	Phosphorylation of RIPK3	Facilitate necroptosis and pyroptosis	(129–131)
	13	AGE/RAGE → MAPK → NF-κB → TNFα/TNFR1 → RIPK3 → MLKL	Upregulation of TNFα and RIPK3	Facilitate necroptosis	(132–137)
	14	ROS → RIPK3 → MLKL	Upregulation of RIPK3	Facilitate necroptosis	(138–141)
Ferroptosis	15	AGE/RAGE → SOD+GPX4 → ROS → lipid peroxidation products	Inhibition of SOD and GPX4	Facilitate lipid peroxidation Facilitate ferroptosis	(142–145)
	16	AGE/RAGE → TF + hepcidin → FPN1	Upregulation of TF and hepcidin Inhibition of FPN1	Increase cellular iron overload Facilitate ferroptosis	(144, 146)
	17	ERK → Nrf2/HO-1 → ROS+TGF-β1	Inhibition of Nrf2/HO-1 Upregulation of ROS Upregulation of TGF-β1	Destroy iron metabolism homeostasis Facilitate ferroptosis Facilitate inflammation	(147–155)
Cuprotosis	18	AGE/RAGE → ATF3 →SPI1 →SLC31A1	Upregulation of SLC31A1	Destroy iron metabolism homeostasis Facilitate cuprotosis	(156–159)

AGE, advanced glycation end products; Akt, protein kinase B; AP-1, activator protein-1; ATF3, activating transcription factor 3; CML, Nϵ-carboxymethyl lysine; ERK, extracellular signal-related kinase; FPN1, ferroportin 1; GPX4, glutathione peroxidase 4; GSDMD, Gasdermin D; HMGB1, high-mobility group box 1; HO-1, heme oxygenase 1; JAK, Janus Kinase; LPS, lipopolysaccharide; MAPK, mitogen-activated protein kinases; MLKL, mixed lineage kinase domain-like; mTOR, mammalian target of rapamycin; NF-κB, nuclear factor kappa-B; NLRP3, Nod-like receptor protein 3; Nrf2, nuclear factor erythroid 2-related factor 2; PI3K, phosphatidylinositol 3 kinase; RAGE, AGE receptor; RIPK3, receptor interacting protein kinase 3; ROS, reactive oxygen species; SIRT1, sirtuins 1; SLC31A1, copper uptake protein 1, also CTR1; SOD, superoxide dismutase; SPI3, transcription factor PU.1; STAT, signal transducer and activator of transcription; TF, transferrin; TGF-β1, transforming growth factor-β1; TLR, Toll-like receptors; TNF-α, tumor necrosis factor-α; TNFR1, tumor necrosis factor receptor 1; VCAM-1, vascular cell adhesion molecule-1.

### Autophagic cell death

Autophagic cell death is characterized by the formation of autophagosomes, followed by phagocytosis and lysosomal degradation (160). Vacuoles are produced due to the4 occurrence of isolated membranes in the cytoplasm of dying cells. These vacuoles accumulate in the cells and eventually mature into autophagosomes, which then fuse with lysosomes (160, 161). This fusion releases relevant enzymes that break down cellular materials, resulting in the autophagy of the cells (161). Essentially, autophagy is a process that recycles macromolecules within cells (162). It is common for cells to activate pro-survival mechanisms rather than trigger direct cell death in response to external stimuli such as starvation, oxygen deficit, and infection at the biological level (162). In such situations, any subsequent cell death that occurs after autophagy cannot be easily attributed to “autophagy-induced” cell death. This is because it is unclear whether the autophagosome itself is enough to rescue the cells from inevitable decompensation (163). In addition, several studies have found autophagic cell death in eukaryotes *in vitro* (164, 165). However, evidence of this type of PCD under *in vivo* conditions is still scarce, and its existence was once doubted (166). Inspiringly, follow-up studies gradually confirmed that cell death can result from autophagy *per se*, and the definition has been preserved (167). The autophagosome is considered a hallmark of autophagy, which is initiated by the effects of phosphatidylinositol 3 kinase (PI3K) and autophagy-related protein Beclin-1 (168). Additionally, autophagy is negatively regulated by the mammalian target of rapamycin (mTOR), which prevents excessive and uncontrolled autophagy (161). Pathological conditions of several diseases such as asthma and tumorigenesis have been identified to be correlated with autophagic cell death (169–172).

### Necroptosis

Cells undergoing necroptosis typically undergo dramatic morphological changes, such as rapid swelling and disruption of the plasma membrane. These changes are markedly different from the mild changes observed in apoptotic cells (173). Despite similar changes, necroptosis is still programmed to be regulated when compared to necrosis (174). Necroptosis also shares several characteristics with apoptosis. It is triggered by tumor necrosis factor receptor 1 (TNFR1) in response to certain stimuli, such as infection and sterile inflammation, with inhibition of caspase-8 of apoptosis (132). The receptor interacting protein kinases (RIPKs) (mainly RIPK1 and RIPK3) are at the center of necroptosis and mixed lineage kinase domain-like (MLKL) (133, 134). The ligation between TNF-α and TNFR1 could result in polyubiquitination of RIPK1, which further assembles the necrosome with RIPK3 and recruits MLKL to promote downstream cell death (135, 136).

The downstream mechanisms of necroptosis consist of the formation of plasma channels and rapid swelling of cells, which usually lead to rapid rupture of the plasma membrane and vigorous stimulation of innate immune responses (175). Necroptosis contributes to the pathogenesis of several diseases. For example, ischemic injury in several organisms, including the brain, myocardium, eyes, and kidneys, has been discovered (176–179). Additionally, the necroptosis of epithelial tissue has been linked to inflammation of the skin and even the pathogenesis of inflammatory bowel diseases (180, 181). Dysfunction of the barrier function is a consequence of necroptosis effects in the pathogenesis (182).

### Pyroptosis

Pyroptosis is a type of PCD that is involved in the innate immune response and is mediated by pro-inflammatory mechanisms, including the effects of multiple inflammasomes (22). Although pyroptosis is dependent on the caspase family, it is typically considered a response to external stimulation such as bacterial infection, leading to the release of a series of pro-inflammatory cytokines and rapid cell death (183), unlike apoptosis, which occurs without inflammation. Caspase-1 is the major enzyme mediating pyroptosis but not apoptosis (184). Classical inflammasomes, such as Nod-like receptor protein 3 (NLRP3), help with the activation of pro-caspase-1, further promoting the perforation of the cell membrane by Gasdermin D (GSDMD), releasing pro-inflammatory cytokines such as IL-1β and IL-18, and finally causing disintegration of cells (26). The activation of NLRP3, mitochondria-producing ROS, and disruption of the lysosomal membrane are significant pathways (185, 186). Other caspases, including caspase-4/5/11, in addition to caspase-1 are also involved in non-classical inflammasome pathways (187). Recently, many studies have focused on the alteration of the above-mentioned key factors in diabetes and comorbidities (188). The pyroptosis of pulmonary epithelium under certain stimulation has also been observed (189, 190).

### Ferroptosis

Ferroptosis is considered a novel type of non-apoptotic PCD. Particularly, it is mainly dependent on cellular iron rather than the key factors in apoptosis such as the caspases family (191). The two lethal incentives of ferroptosis were considered overloading of iron and excess accumulation of lipid peroxides (192).

As an essential nutritional element, iron is absorbed by the intestinal epithelial cell and stored in the form of ferric ion (Fe^3+^), combined with transferrin (TF), and involved in transmembrane transportation (193, 194). The pH value influences the release of Fe^3+^, which is reduced to ferrous iron (Fe^2+^) after release from TF under an acidic environment and further participates in downstream reactions (195). Under normal circumstances, the level of intracellular iron is maintained in homeostasis (196). While under certain pathogenic states, dysfunction of iron metabolism may produce overloaded iron and result in the production of hydroxyl radical and oxidative stress response due to a reaction between lipid peroxide and free Fe^2+^ (197). Fe^2+^ also promotes other metabolic processes, contributing to the production of more lipid ROS (195). The increased level of oxidative stress again enhanced the process of ferroptosis (195, 196). In a word, the metabolic dysfunction aggravates oxidative stress, which in turn results in ferroptosis and tissue damage. Such pathogenic states may further exacerbate under the hyperglycemic environment, which is characterized by metabolic dysfunction and imbalance of the oxidant-antioxidant system, leading to more generation of lipid peroxides (198). High blood sugar levels facilitate ferroptosis in diabetic complications such as myocardial ischemia and diabetic retinopathy (199, 200). Diabetic pneumopathy could also be predicted. In addition, the inhibition of lipid antioxidants such as glutathione peroxidase 4 (GPX4) would further decrease elimination of lipid peroxide, which also contributes to occurrence of ferroptosis (192).

### Cuprotosis

Along with ferroptosis, other cell deaths dependent on heavy metals have been reported, including copper-induced cell death. This is a brand-new type of PCD, which differed from ferroptosis and necroptosis closely relating to oxidative stress, and has been named “cuprotosis” or “cuproptosis” (113). Though copper *per se* is an essential coenzyme factor, intracellular copper concentrations are still maintained at an extremely low level to prevent toxicities to cells (201). However, under certain pathological conditions, such as diabetes, copper metabolisms could be abnormal, leading to a higher concentration of circulation copper (156). This is possible due to the over-produced AGEs activating transcriptional factors, specifically transcription factor 3 (ATF3) and transcription factor PU.1 (SPI1), following the upregulated expression of the copper transmembrane transporter high-affinity copper uptake protein 1 (CTR1, also SLC31A1) and increased intracellular Cu concentration (157). The homeostasis of copper is then destroyed. Furthermore, ferredoxin 1 (FDX1), the mitochondrial reductase, was identified to reduce Cu^2+^ to Cu^+^ in the mitochondria, promoting more ROS to be released and ultimately cuprotosis of tissue cells (158). In fact, studies on copper-related cell death are only at an early stage (159). We are looking forward to more mechanisms of cuprotosis being explored.

## Interactions of multiple forms of non-apoptotic PCDs in diabetic pulmonary dysfunction

When it comes to non-apoptotic PCDs, recent studies have shown that AGEs may directly induce ferroptosis of tissue cells and promote the release of iron, destroying iron homeostasis (202). Consequently, dysfunctional organs including the lung resulted, and downstream oxidative stress response was also activated (203, 204). Moreover, pyroptosis, necroptosis, and ferroptosis are involved to a certain extent.

### Interactions of autophagic cell death

Although divergences still exist, a consensus has been reached that autophagic cell death is a type of non-apoptotic PCD that relies on the formation of autophagosomes and the occurrence of autophagy (161). Previous studies have shown an increase in autophagy and activation of autophagic proteins in epithelial cells in pulmonary diseases such as COPD, and idiopathic pulmonary fibrosis was also observed (205, 206). Relevant signaling pathways mainly include class III PI3K/Beclin-1 and class I PI3K/Akt/mTOR, while recent studies have discovered the effects of AGEs acting on both of them (114). However, the class III PI3K/Beclin-1 can add a number of autophagic vacuoles and facilitate autophagy, promoting cell injuries under AGE administration, while class I PI3K/Akt/mTOR mainly manifested as a negative regulator of autophagy and can be inhibited by the AGE–RAGE interaction (114). The total effects turn out to be an exacerbation of autophagic cell death. AGE–RAGE interaction can also directly mediate the inactivation of mTOR, the final negative regulator of the classical pathway, which further facilitates the autophagy of tissue cells. Ma, J. et al. revealed that influenza virus infection, such as H5N1, could cause autophagic cell death by suppressing mTOR and do serious damage to pulmonary function, even acute respiratory distress syndrome (ARDS) (115). These studies have identified that the intensified AGE–RAGE interaction in diabetes can further intensify this process of autophagic cell death.

**Figure 1:**
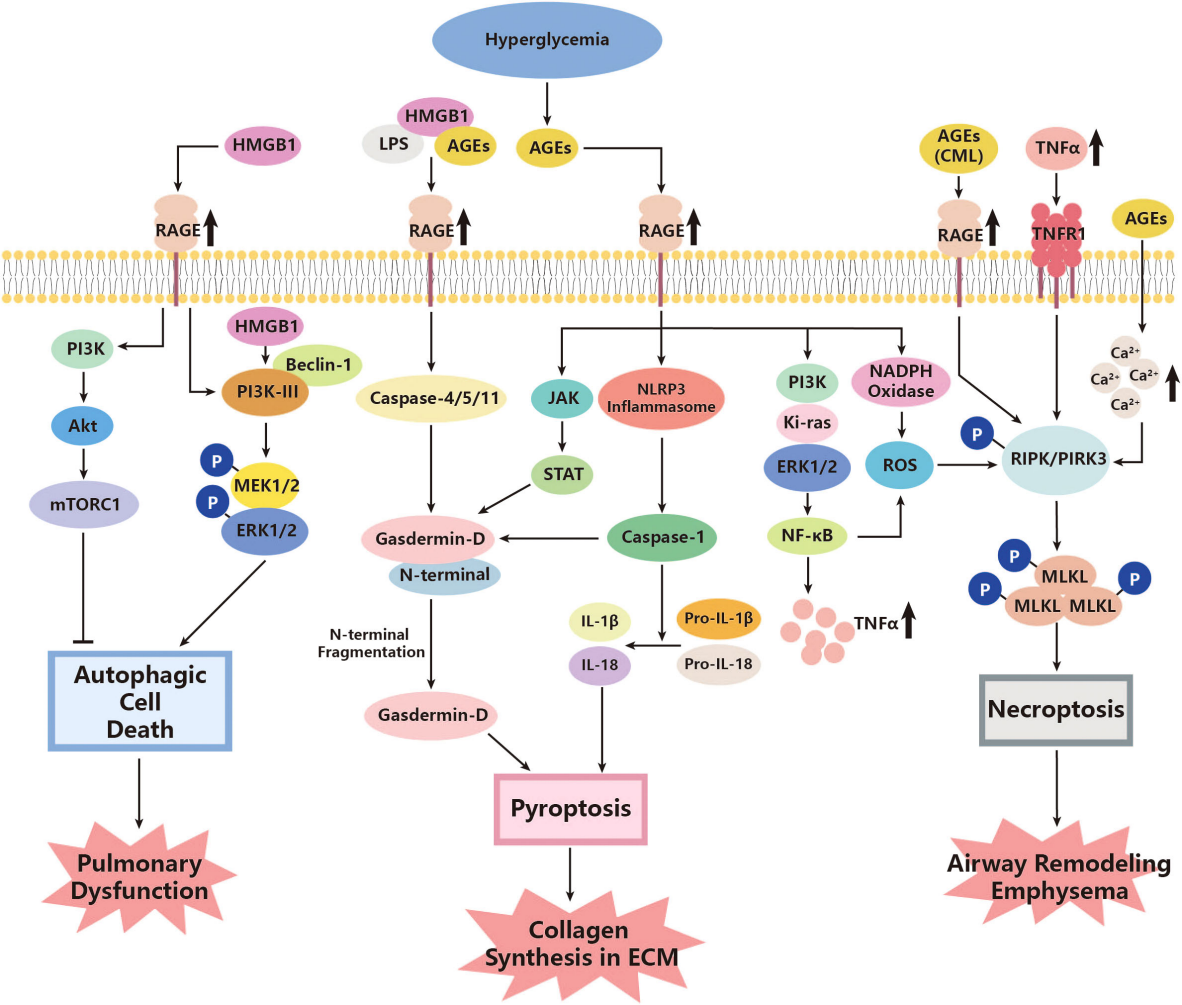
Roles of autophagic cell death, pyroptosis, and necroptosis in the pathogenesis of diabetic lung disease. AGEs, advanced glycation end products; Akt, protein kinase; CML, Nϵ-carboxymethyl lysine; ERK, extracellular signal-related kinase; HMGB1, high-mobility group box 1; JAK, Janus Kinase; Ki-ras, K*irsten rat sarcoma viral oncogene;* LPS, lipopolysaccharide; MEK, mitogen-activated protein kinases (MAPK) kinases; MLKL, mixed lineage kinase domain-like; mTORC1, mammalian target of rapamycin C1; NADPH, Nicotinamide Adenine Dinucleotide Phosphate Oxidase; NF-κB, nuclear factor kappa-B; NLRP3, Nod-like receptor protein 3; PI3K, phosphoinositide 3 kinase; PIPK, receptor interacting protein kinases; RAGE, AGE receptor; ROS, reactive oxygen species; STAT, signal transducer and activator of transcription; TNF-α, tumor necrosis factor-α; TNFR1, TNFα activating TNF receptor 1.

There seems to be some ambiguity regarding the regulation of mTOR by PI3K and protein kinase B (Akt). The classical PI3K/Akt/mTOR signaling pathway can inhibit autophagy, as mentioned above (207). However, another research study on rats has suggested that autophagy may be abnormally attenuated in diabetes, where a higher level of PI3K/Akt/mTOR and a low level of Beclin-1 are observed, and inhibiting the PI3K/Akt/mTOR pathway could help with promoting apoptosis and reducing tissue pathology (208). Nevertheless, the exact roles of autophagic cell death in diabetes complications remain unclear, especially with regard to pathogen clearance and the prevention of infection spread (116). The overproduced ROS could possibly express the relevance. Previous research has indicated that autophagy can reduce pathogen burden and facilitate adaptive immunity (209). However, increased ROS levels can facilitate the synthesis of inflammasome, followed by upregulating mTOR and leading to inhibition of normal autophagy (116). Given the overproduction of ROS in diabetes, normal autophagy could be impaired, further reducing the ability of organs to defend against infection, including the lungs (116). These findings highlight the complexity of the regulatory effects of PI3K, mTOR, and even autophagic cell death on pulmonary function in diabetes (**Figure 1**).

On the whole, studies on autophagic cell death in relation to lung diseases and diabetes were relatively limited. Therefore, more attention needs to be given to this field in the future, especially the multifaceted effects of this unique form of non-apoptotic PCD.

### Interactions of pyroptosis

Multiple roles of pyroptosis have been identified in pulmonary injury related to diabetes. One of the major pathways that show such associations turns out to be AGE–RAGE (117, 118). As a significant receptor in diabetes signaling, RAGE has also been found to be involved in pyroptosis and do harm to the lungs. According to a study by Yang J et al., under specific external stimuli such as hemorrhagic shock (HS), the release of high-mobility group box 1 (HMGB1) can be induced (117). It further interacts with RAGE to enter the pulmonary endothelial cells, triggering the formation of pyroptosome and activation of caspase-1 (117). Given the activation of RAGE and signaling of HMGB1 in diabetes, this could be a possible pathway for initiating pyroptosis of pulmonary endothelial cells and the pathogenesis of angiopathy in the lungs (119). Moreover, caspase-1, which is elevated in diabetes, cleaves SIRT1 to upregulate activator protein-1 (AP-1), ultimately leading to an increase in VCAM-1 of endothelial cells, promoting adherence of monocytes (120). Another crucial factor involved in pyroptosis is caspase-11, which has also been identified to play a role in this process (121). Researchers have found that HMGB1 and lipopolysaccharide, an endotoxin, can form a complex and internalize into the endo-lysosome of endothelial cells with the assistance of interaction with RAGE. This complex can further activate caspase-11 (121). The mature caspase-11 thus can directly activate caspase-1 without NLRP3 and promote pyroptosis (122). Regarding NF-κB, it has also been observed to increase its expression in diabetes, converting pro-caspase-1 to mature caspase-1 and facilitating pyroptosis of type I alveolar epithelial cells through Toll-like receptors (TLRs) and NLRP3 pathways (123). Furthermore, upregulation of both caspase-1 and caspase-11 was found to accelerate the cleavage of pro-IL-1β and pro-IL-18, promoting the release of mature IL-1β and IL-18, the inflammatory cytokines, which further participate in downstream pathogenesis (122, 124). In addition, a novel study has suggested that added AGEs could bind with HMGB1–lipopolysaccharide (HMGB1-LPS) complexes, forming a triplet and exacerbating the above-mentioned mediating RAGEs (84).

In diabetes, high glycemic levels can promote the production of ROS, which mainly originates from the endoplasmic reticulum (ER) stress, damaged mitochondria, and upregulated NADPH (210, 211). The increased oxidative stress, reflected in the growing production of ROS, may further exacerbate pyroptosis and relevant inflammation processes in the lung, especially endothelial dysfunction (210, 212). Heid ME et al. identified that ROS could activate NLRP3 inflammasome and increase the expression of caspase-1, hence facilitating proptosis and IL-1β release (128). The inflammatory cytokines were then released from the pyroptotic cells through the membrane pore made by the N-terminal domain (NTD) of GSDMD, which was cleaved by mature caspase-1 (212). Interestingly, the GSDMD protein was found to be regulated by the JAK/STAT pathway (152). Wenyi Z. et al. observed that the expression of GSDMD significantly decreased under treatment with the f JAK/STAT inhibitor, which was upregulated in response to AGE–RAGE action as mentioned above (97, 213). In addition, the largely released pro-inflammatory cytokine, IL-1β, could enhance the effects of ECM in human bronchial epithelial cells induced by TGF-β, thereby intensifying the pulmonary structural alterations and functional abnormalities mentioned above (126). *In vitro* experiments also support these effects. Yang F. et al. have recognized that downregulating caspase-1 in cells treated with high levels of glucose could strongly inhibit pyroptosis of the cells, decrease the release of IL-1β and TGF-β, and further relieve pathogenic processes such as collagen synthesis and ECM, which were due to the activation of TGF-β (127).

This evidence showed the possibility that pyroptosis could contribute to pulmonary dysfunction by means of cross-linking with the abnormal metabolic, oxidative stress, and inflammation pathways under the hyperglycemia condition of diabetes (**Figure 1**).

### Interactions of necroptosis

Several studies have identified that diabetes may be an important factor in inducing necroptosis in certain tissues through several classical pathways. Fiuza C. et al. found that AGE/RAGE could help with activating NF-κB and further facilitate TNF-α secretion in microvascular endothelial cells (137). The TNF-α/TNFR1 then stimulates downstream cell necroptosis through RIPK and MLKL, as described above (132–136). Yang et al. observed that Nϵ-carboxymethyl lysine (CML), a major member of advanced glycation end products, promotes the phosphorylation of RIPK3 and further facilitates necroptosis of tissue cells through interaction with RAGE (130). Although CML is expressed at a low level in normal physiological conditions, it can be upregulated under pathological conditions, such as diabetes. CML can bind with factors such as HMGB1, leading to the activation of TLRs and NF-κB, and even pyroptosis, as mentioned in the last section (129). HMGB1 is actually a type of damage-associated molecular pattern (DAMP) released from necroptotic cells such as airway epithelial cells (131). Necroptosis has also been found to positively interact with ROS, and such effects could intensify in diabetes (138). A high concentration of glucose has been observed to upregulate RIPK3 in tissue cells *in vitro*. Inhibiting the necroptosis of these cells decreased ROS production, while treating cells with ROS scavengers, in turn, decreased RIPK3 levels (138). These pieces of evidence indicate that the diabetic condition could not only directly promote necroptosis of tissue cells but also release pathological factors from necroptotic cells, further resulting in downstream reactions. What is worth mentioning is that necroptosis may co-exist with other types of CTDs, especially pyroptosis. In those with pulmonary dysfunction, including COPD or asthma, RIPK3 was shown to increase in the lung and airway tissues (139, 140), indicating the pathogenic effects of necroptosis in lung damage. Moreover, there is research also identifying attenuating RIP3K as well as the downstream MLKL, which can help with relieving airway remodeling and emphysema, which ameliorates pulmonary function to a certain extent (141) (**Figure 1**).

### Interactions of ferroptosis

Previous studies showed that ferroptosis was related to ischemia-reperfusion injury (IRI)-relevant diseases (200). Under the high level of blood glucose in diabetes, vascular diseases were found in multiple systems and organs rich in blood supply, such as the cardiovascular system, optical system, and renal system (199, 214). As mentioned before, the lung has an abundant capillary network; thus, it might be inferred that the lung tends to be damaged in diabetes, including the pathways of ferroptosis. Moreover, the core mechanisms of ferroptosis, containing over accumulation of lipid peroxides as well as the overproduction of ROS, were found to play roles in the pathogenesis of several pulmonary diseases (146, 191). Combining the abnormal metabolic dysfunction and oxidative stress in diabetes, the pulmonary dysfunction due to ferroptosis under the corresponding circumstances could be significant to a certain extent.

Ferroptosis is a type of PCD that is dependent on ROS (215). In diabetes, there is an increase in ROS production in cells (142), which can lead to lipid peroxidation and further enhance ferroptosis (143). However, the effects of antioxidant agents are blocked under diabetic conditions. GPX4 is a powerful antioxidant enzyme that can inhibit lipid peroxidation and prevent ferroptosis. Previous studies have shown that GPX4 significantly decreases in certain types of pulmonary diseases such as lung fibrosis (147, 148) and tuberculosis (216). Recently, researchers have also found that diabetes can alter the levels of GPX4 (144). High blood glucose levels can increase the expression of RAGE and its ligands (145). According to Li YJ et al., overexpression of RAGE is associated with the suppression of superoxide dismutase (SOD), which inhibits the antioxidant pathway mainly mediated by GPX4. This leads to an increase in lipid peroxidation products and ferroptosis of tissue cells (144). Moreover, the same team has also revealed the possible effects of upregulated RAGE on iron uptake and storage. An increase in serum RAGE levels is significantly associated with a higher level of TF, which symbolizes more iron being transported into cells. It also decreases the export of iron through the elevation of hepcidin, which inhibits the iron exporter ferroportin1 (FPN1) (144). These all could contribute to cellular iron overload and exacerbate ferroptosis (146).

**Figure 2:**
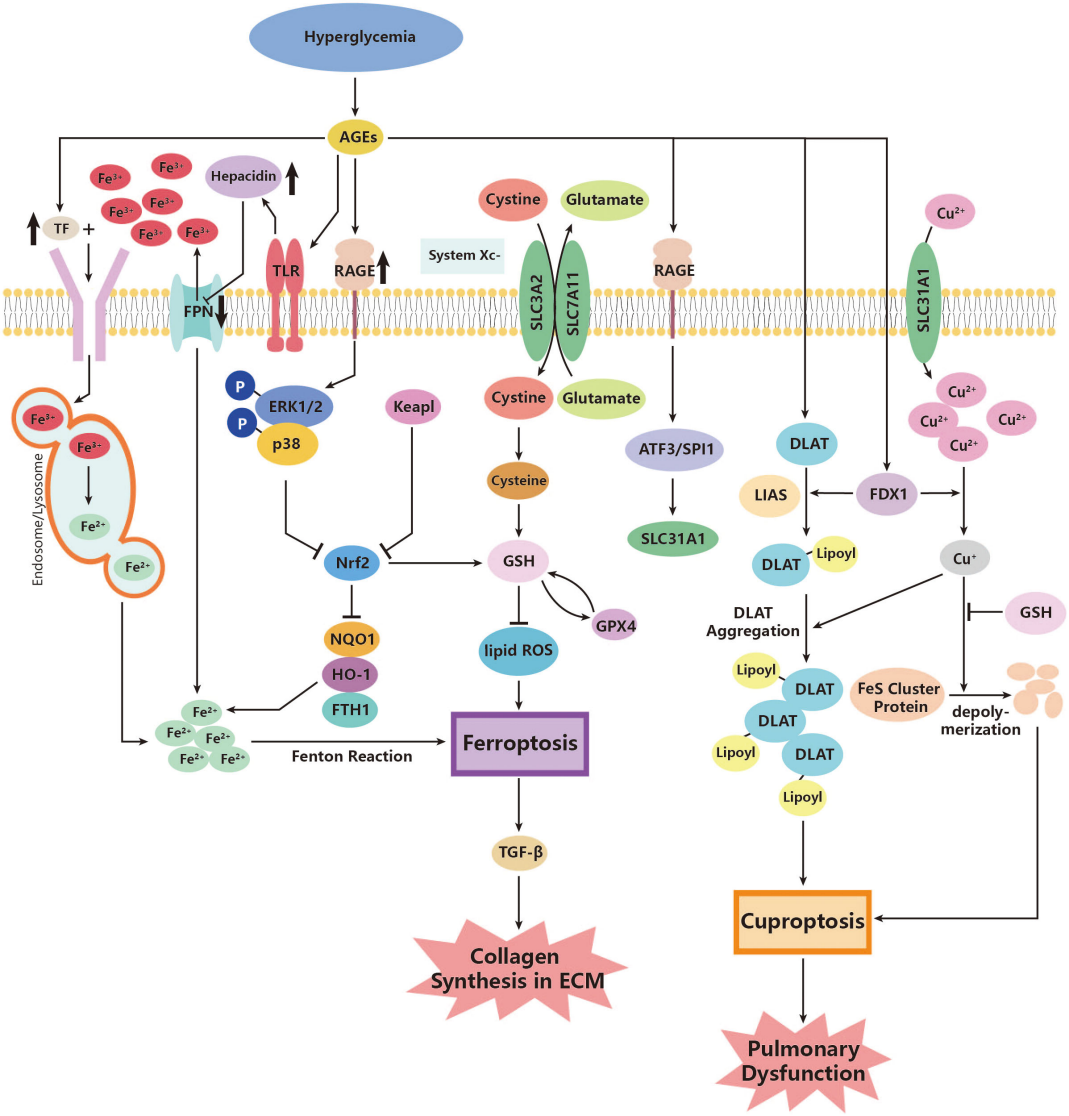
Roles of ferroptosis and cuproptosis in the pathogenesis of diabetic lung disease. AGEs, advanced glycation end products; ATF3, activating transcription factor 3; DLAT, *dihydrolipoyl transacetylase;* FDX1, ferredoxin 1; FPN, ferroportin; FTH1, ferritin heavy chain 1; GPX4, glutathione peroxidase 4; GSH, glutathione; HO-1, heme oxygenase 1; Keapl, Kelch-like ECH-associated protein 1; LIAS, lipoyl synthase; NOQ1, NADH dehydrogenase, quinone 1; Nrf2, nuclear factor erythroid 2-related factor 2; p38, p38 mitogen-activated protein kinases; RAGE, AGE receptor; ROS, reactive oxidative species; SLC3A2, *Solute Carrier Family* 3, Member 2; SLC7A11, *Solute Carrier Family* 7, Member 11; SLC31A1, high affinity copper uptake protein 1; SPI1, transcription factor PU; System Xc-, cysteine/glutamate transporter receptor; TF, transferrin; TGF-β, growth factor-β; TLR, Toll-like receptor.

Apart from GPX4, the nuclear factor erythroid 2-related factor 2 (Nrf2) is another regulatory factor that functions as an antioxidant. Nrf2 defends against the damage caused by overloaded oxidative stress and inhibits ferroptosis (149). As a transcription factor for cellular antioxidants, Nrf2 is released in response to the production of ROS and promotes the production of more antioxidants (150). One significant downstream antioxidant is heme oxygenase 1 (HO-1), which promotes the catalyzation of heme, producing ferrous iron (151). The Nrf2/HO-1 pathway contributes to the homeostasis of iron metabolism and plays anti-inflammatory and antioxidant roles in cells (152). However, this defense system has been revealed to be destroyed in patients with pulmonary dysfunctional diseases such as lung fibrosis, COPD, and asthma (147, 153, 217). Interestingly, researchers have discovered the downregulation of Nrf2/HO-1 in DM patients (154), probably through the activation of extracellular signal-related kinase (ERK) (155). Therefore, it is reasonable to assume that diabetes could exacerbate ferroptosis in the lung and further contribute to the pathogenesis of pulmonary dysfunction. Additionally, downstream inflammation is also worthy of attention. Researchers found overexpression of TGF-β1 in lung fibrosis due to the inhibition of Nrf2 during ferroptosis. Therefore, ferroptosis can be a significant source of TGF-β1 under diabetic oxidative stress and do damage to the lung (148) (**Figure 2**).

### Interactions of cuprotosis

Current studies on cuprotosis have focused on tumor cells and the life expectancy of relevant cancer diseases in various organs such as hepatocellular carcinoma, lung adenocarcinoma, clear cell renal carcinoma, and pancreatic cancer (218–221). Still, earlier studies that found abnormal copper metabolism in patients with diabetes compared to healthy controls demonstrate that cuprotosis may also play roles in several diabetic complications (222, 223). Few studies have mentioned the possible contribution of cuprotosis to diabetic pulmonary dysfunction; however, in view of cuprotosis often happening in cells with abundant mitochondria and energy consumption, the vascular endothelial cells of the lung, which are exposed directly to high concentrations of oxygen in gas exchange, may be potential targets of cuprotosis, especially in a pathological state like diabetes (224). In research using mouse experiments, researchers have found that an abnormally increased copper ion content can lead to higher production of ROS and decrease SOD expression in pulmonary tissue (225). Thus, it is reasonable to assume that pulmonary damage may be due to exacerbated cuprotosis in diabetic conditions. The above-mentioned signaling pathway, ATF3/SPI1/SLC31A1 (157), could be a possible pathway in diabetic lungs, though no direct studies have figured out the exact effect. Relevant studies can be a new way of studying non-apoptotic cell death effects in pulmonary dysfunction, not only cancer (**Figure 2**).

### Possible drugs for targeting NAPCDs in pulmonary dysfunction in DM

Though specific targeted drugs have not been largely studied or produced, some medicines are still found to contribute to influencing NAPCD-related pathways, leading to pulmonary dysfunction in DM. According to Huang et al., they have proved in mice that the drug, hispolon, can provide protection for pulmonary cells through inhibition of PI3K/Akt/mTOR, further resisting inflammation and oxidative stress under diabetic environments (226). Moreover, the chemical ingredients such as syringic and ascorbic acids, as well as nobiletin, have also been found to suppress autophagic cell death by downregulating the PI3K/Akt/mTOR pathway to prevent possible injuries to the lungs (227, 228). As for pyroptosis, several small molecules targeting the essential sites have already been developed to deal with conditions of multiple diseases. Possible molecules include MCC950, 3,4-methylenedioxy-β-nitrostyrene (MNS), and oridonin, which block NLRP3 and further play roles in DM and pulmonary injuries (229). Furthermore, necrosulfonamide and disulfiram have been identified to attenuate GSDMD to prevent LPS-induced lung fibrosis and pneumonia (229). Furthermore, there have been studies showing that blocking RIPK1, RIPK3, and MLKL can therefore improve the pulmonary function in diseases such as COPD and ARDS, as well as insulin resistance (230–232); the inhibitors include GW806742X and Nec‐1 (230, 232). Additionally, considering that ROS plays a significant role in the initiation of ferroptosis, vitamin E, which has weak chemical bonds, can help with trapping radicals and therefore protect the pulmonary from ferroptosis as well as oxidative damage under diabetic conditions (233, 234). Cuprotosis is a relatively novel concept. Though exact inhibitors have been rarely reported, they can be promising directions for future studies on diabetic pulmonary dysfunction.

### Non-apoptotic programmed cell death in diabetics with COVID-19

SARS-Cov-2 has become a newly generated source of infectious pulmonary damage since the explosion of COVID-19. According to recent large-scale studies, diabetes is a significant risk factor for COVID-19, adding to the severity of the disease and mortality of the patients (235). During the process, non-apoptotic cell death has been proven to play a certain role. The most-mentioned one turned out to be ferroptosis. Bost, Pierre et al. found that the monocytes can promote the expression of cytokines and duplication of SARS-Cov-2, further ROS production in the mitochondria of endothelial cells under high-glucose level conditions in diabetics (236). Moreover, Jankauskas et al. revealed that the overproduced ROS exacerbates lipid oxidation and enhances ferroptosis, manifesting as increased levels of markers of ferroptosis, such as GPX4 (237). This can be a possible non-apoptotic cell death-mediated pathway for pulmonary damage in COVID-19 patients with diabetes. Furthermore, considering diabetes as a metabolic syndrome, the accompanying pro-inflammatory effects facilitate more production of inflammasome NLRP3, therefore adding to the pyroptosis of tissue cells in the lungs of COVID-19 patients (238, 239). Inhibition of NLRP3 by certain drugs can largely relieve the cytokine storm (240, 241).

## Summary and perspectives

This article provides a brief review of the traditional pathogenesis of diabetes pulmonary disease (DPD), various forms of NAPCD, and molecular regulatory pathways. Building on the foundation, it introduces the potential role of NAPCD in DPD.

Cell death plays a crucial role in the development and homeostasis of nearly all multicellular organisms. While apoptosis was once considered the primary pathway regulating cell death, recent years have witnessed the discovery of several forms of regulated cell death associated with inflammation, degenerative diseases, and cancer. This deepened understanding of regulated NAPCD has shed new light on the targeted treatment of these diseases. Inducing NAPCD has received increasing attention in the field of tumor therapy. For instance, in lung cancer studies, inhibitors of gamma-glutamyl cysteine ligase, glutathione, SLC7A11, and activators of iron have demonstrated anti-cancer effects by triggering ferroptosis. Some small molecules that target these pathways are now under consideration as candidate drugs for clinical trials. Despite diabetes having a lower mortality rate compared to tumors, diabetic complications impact vital organs such as the kidneys, eyes, and nervous system, significantly affecting patients’ quality of life. Presently, the role of NAPCD in the pathogenesis of diabetes is still in its infancy. For example, necroptosis can contribute to insulin resistance and damage to retinal, myocardial, and renal cells by mediating necrotizing inflammation, thereby influencing the onset and progression of diabetes and its associated chronic complications. Inhibitors targeting necroptosis are currently under development for various disease models, such as neurodegenerative diseases, acute heart failure, acute renal failure, systemic inflammation, and COPD. Consequently, the exploration of NAPCD in diabetic complications offers novel insights into the prevention and treatment of diabetes and its related health challenges.

## Author contributions

All listed authors have made a substantial and direct contributions to this work. CW and QW conceptualized the review. YD and SZ are responsible for literature collection and article draft. LQ, ZP, and JZ are responsible for literature collection. DX, ML, XZ, and QW guided writing and revised the manuscript. All authors contributed to the article and approved the submitted version.
